# Dr. Swan M. Burnett

**Published:** 1872-10

**Authors:** 


### PART III.

### Editorials, Correspondence and Miscellaneous.

### DR. SWAN M. BURNETT,

Gives our readers one of his splendid papers upon the "Functional Diseases of the Eye." The paper, in this number, upon Myopia, will be read with great interest. The valuable thoughts and suggestions therein contained, should be treasured up by our readers. We hope Dr. Burnett will continue his valuable papers. He is certainly one of the ablest writers, upon his specialty, in this country.

